# Unveiling the Potential of Plant-Derived Diarylheptanoids and Their Derivatives in Bio-Based Polyurethane Compositions

**DOI:** 10.3390/plants14050775

**Published:** 2025-03-03

**Authors:** Matiss Pals, Jevgenija Ponomarenko, Maris Lauberts, Lilija Jashina, Vilhelmine Jurkjane, Alexandr Arshanitsa

**Affiliations:** Latvian State Institute of Wood Chemistry, Dzerbenes Street 27, LV-1006 Riga, Latvia; matiss.pals@kki.lv (M.P.); jevgenija.ponomarenko@kki.lv (J.P.); lilija_jasina@inbox.lv (L.J.); ligms@edi.lv (V.J.); alexandr.arsanica@kki.lv (A.A.)

**Keywords:** diarylheptanoids, black alder (*Alnus glutinosa*) bark, bio-based polyurethane, biodegradation, rigid polyurethane foam, thermal oxidative aging, flame retardancy

## Abstract

The key challenge in polymer science is developing sustainable synthesis methods using renewable feedstocks. This study explores plant-derived diarylheptanoids with various structures as the building blocks for polyurethane (PU) materials. Diarylheptanoid glucosides isolated from black alder (*Alnus glutinosa*) bark were hydrolyzed and fractionated to remove sugar moieties. The resulting diarylheptanoids, along with unhydrolyzed analogues and curcumin, were used as biomass-based polyols to synthesize model PU films. Incorporating diarylheptanoids enhanced the mechanical strength and reduced the flexibility of PU due to increased crosslinking, with effects proportional to the OH functionality of the biomass-based polyols. Weight loss, FTIR, and Py-GC-MS/FID analyses revealed that the catechol moieties and the glucosidic bonds are biodegradable structural subunits of diarylheptanoids incorporated into PU films. Rigid polyurethane foams (PURs) incorporating high-OH-functionality diarylheptanoid glucosides such as oregonin demonstrated significantly higher compression strength and less weight loss during non-isothermal thermal analysis in air compared to those of commercial polyol-based foams. A cone calorimeter test showed that the PUR foam with diarylheptanoid derivatives had a lower degradation rate, a longer flame-burning time, 30% less heat emission, and 25% less smoke, indicating improved flame retardancy. Adding 1–2% oregonin-enriched black alder bark extracts to commercial Elastopir 1132/509/0 PUR foam significantly improved its resistance to thermal oxidative aging, outperforming the commercial antioxidant Irganox.

## 1. Introduction

Today, and in the near future, the key challenge in polymer science is developing alternative methods for polymer synthesis that prioritize renewable feedstocks, while advancing efficient recycling technologies. Due to their remarkably broad range of applications, polyurethanes (PUs) rank among the most widely produced synthetic polymeric materials, with the total production volume projected to reach 29.3 million tons by 2029 [1]. Chemically, polyurethanes are polymeric carbamates defined by the presence of urethane bonds (-NHCOO), which are formed through a polyaddition reaction between monomers containing two or more hydroxyl groups (diols/polyols) and di- or multifunctional isocyanates. Modifications to diol/polyol backbones can be readily achieved through various chemical methods, enabling the creation of a diverse array of polyurethane materials with a broad spectrum of physicochemical properties that can be finely adjusted. Lupranol polyols, produced by BASF, are an example of a commercial polyol extensively used in the synthesis of polyurethane materials. These polyether polyols are derived from petroleum-based raw materials, primarily propylene oxide and ethylene oxide. The production of other commercial polyether polyols also relies on these oxide intermediates. Similarly, polyester polyols are synthesized from fossil-based feedstocks, such as phthalic anhydride, adipic acid, toluene, and benzene, with the latter two being the key components in aromatic polyester polyols. To address growing environmental and resource sustainability concerns, fossil-derived polyols in PU compositions can be partially or fully replaced by biomass-based polyols, the polyols from vegetable oils (biopolyols), and the polyols from chemical recycling (repolyols). The latter group includes recycled polyethylene terephthalate (e.g., Terol^®^ by Huntsman) and post-consumer recycled polyols sourced from waste polyurethane foam (e.g., Renuva^TM^ by Dow). Additionally, CO₂-based polyols (e.g., Cardyon^®^ by Covestro) utilize carbon dioxide as a feedstock, offering another innovative pathway toward sustainable polyurethane production [2,3,4,5].

Among the various sources of biomass-based polyols, tree bark—a by-product of the forestry and wood-processing industries—is an abundant and underutilized resource. Different technologies are being developed for the production of bark-derived precursor chemicals for the synthesis of polyurethane materials [6,7,8,9]. The extraction of bark under appropriate conditions yields bark-based products containing hydroxyl (OH) groups of varying origin. The previous studies have demonstrated that the extraction of black alder (*Alnus glutinosa*) bark using fast, energy-efficient microwave-assisted extraction with water at 70–90 °C produces a mixture of polyols dominated by diarylheptanoids. These polyols contain 15.1 mmol·g^−1^ of total hydroxyl groups reactive with diphenylmethane diisocyanate, making them suitable for polyurethane (PU) synthesis [10,11,12,13,14,15]. Diarylheptanoids are secondary metabolites found in significant quantities in the bark of various tree species, particularly those belonging to the *Betulaceae* family, such as alder trees, which are common in Latvia. These compounds are characterized by two aromatic rings connected by a seven-carbon chain. Typically studied for their biological activities in small-scale applications, these compounds have untapped potential for broader utilization. Our approach is original in its innovative use of black alder (*Alnus glutinosa*) diarylheptanoid derivatives as building blocks for the synthesis of bio-based rigid polyurethane foams, leveraging their unique structural properties, such as their richness in aromatic segments; flexible, saturated carbon chains; and the presence of 5–8 phenolic and aliphatic hydroxyl (OH) groups per diarylheptanoid unit [16]. These features are expected to promote the formation of branched and extensively crosslinked PU networks, resulting in a rigid matrix [17]. Rigid polyurethane (PUR) foam is highly valued across industries, such as construction, aerospace, and automotive [18,19,20]. The construction sector, in particular, drives significant demand for PUR foam due to its superior thermal insulation properties. Effective insulation helps reduce energy consumption, lower infrastructure costs, and meet sustainability standards. Thermal insulation is a critical global priority, as heating and cooling buildings account for one-third of annual worldwide energy consumption [21]. With thermal conductivity ranging from 0.025 to 0.046 W·m^−1^·K^−1^, PUR foam outperforms materials like mineral wool, polystyrene, and cellulose [22]. Although recent research has prioritized natural insulation materials, such as cellulose, straw, and hemp, these materials are susceptible to moisture and fire, which limits their applicability in certain settings [23,24]. In line with the growing emphasis on sustainability within the construction industry, bio-based alternatives to PUR foams derived from renewable resources such as vegetable oils have been developed. While oils like soybean and palm have been widely used, they are often included in human food supply, emphasizing the need for non-edible and abundant alternatives such as tree bark.

This study explores the potential of alder bark-derived diarylheptanoid derivatives with diverse structures as building blocks for polyurethanes (PUs), with a particular focus on rigid polyurethane foams. Among the plant-derived diarylheptanoids, curcumin—commonly found in plants of the *Zingiberaceae* family, including *Curcuma longa*—is the only compound extensively studied as a component of PU materials. Polyurethanes incorporating curcumin have been synthesized for applications in various fields, including medicine, food packaging, coatings, textiles, and sensors. However, curcumin has not yet been applied in the synthesis of rigid polyurethane foams [25,26,27]. This limitation can be attributed to the relatively low hydroxyl (OH) functionality of curcumin, which contains two aromatic ortho-methoxyphenol groups and an α,β-unsaturated β-diketone group. In this context, oregonin, an alder bark-derived diarylheptanoid xyloside having three aliphatic and four phenolic OH groups, presents a more promising alternative. In our previous studies, it was shown that it can act as a crosslinker for the PU network, and methods for incorporating oregonin-based polyols into polyurethane foams were developed [10,11,12,15].

This study, for the first time, examines how the structural properties of diarylheptanoids—specifically the presence of sugar moieties and variations in aromatic ring structures (e.g., methoxylated vs. catechol moieties)—influence the mechanical performance and biodegradation behavior of PU materials. Furthermore, it investigates the impact of using alder bark-derived diarylheptanoids as the building blocks for rigid PU foams on their combustion behavior, providing a novel contribution to the field. Combustion behavior, particularly flammability, is a critical factor for the successful application of bio-based polyurethanes in construction, furniture, and transportation, where fire safety is the primary concern [28,29].

Recognizing the phenolic hydroxyl groups in the structures of plant-derived diarylheptanoids, their potential as antioxidants was also investigated to enhance the thermo-oxidative resistance of PU materials. Thermo-oxidative resistance is a fundamental property of the PU matrix, determining its stability during long-term exposure to elevated temperatures and influencing its combustion behavior [30]. Thermal degradation, which occurs via a free-radical chain mechanism, can result from thermal processing or usage conditions. For example, degradation may arise from infrared radiation absorption, typically from solar spectra or nearby heat sources. Thermal and mechanical stresses can facilitate the scission of polymer chains into macroalkyl radicals, initiating the formation of hydroperoxides and subsequent degradation. Various radical scavengers, including hindered phenols, are commonly used to protect PU materials against thermo-oxidation. Low-level volatility is a crucial property of antioxidants for PU systems, as it prevents their loss from the material at elevated temperatures [31,32]. Replacing synthetic antioxidants with plant-derived phenolic components aligns with modern bioeconomy trends [33]. Due to their chemical structures, alder bark-derived diarylheptanoids exhibit a lot of radical-scavenging activity and can compete with commercial antioxidants [34]. The sugar moieties in diarylheptanoid derivatives, connected to the phenolic part via glycosidic bonds, enable their incorporation into the PU matrix. The reactive aliphatic hydroxyl groups of the sugar units interact with isocyanates, while the less reactive phenolic hydroxyl groups remain available for radical scavenging. Controlling the reactivity of various OH groups of diarylheptanoids in urethane-forming reactions can be achieved by the use of various catalysts [11]. By chemically bonding the antioxidant to the PU network, its removal during heating is prevented, maintaining its efficiency under high-temperature conditions.

This study aims to evaluate the alder bark-derived diarylheptanoid derivatives as bio-based components for the synthesis of rigid polyurethane (PUR) foams. The objectives include the extraction, chemical modification, and characterization of diarylheptanoid-based polyols, followed by their application in the production of model polyurethane films. Through comparative analyses of the mechanical properties and the durability of polyurethane films derived from diarylheptanoid-based polyols with various structures, the most promising diarylheptanoids were selected for the synthesis of rigid polyurethane foams, which were subsequently subjected to comprehensive characterization. The findings demonstrate that the alder bark diarylheptanoid glucosides containing catechol moieties and aliphatic hydroxyl groups are promising building blocks for increasing the mechanical strength and reducing the flammability of PU foams. Additionally, these compounds were identified as potential technical antioxidants for commercial urethane material production. By exploring the utilization of tree bark-derived diarylheptanoids as biomass-based polyols, this research advances the development of bio-based polyurethanes and underscores the potential of forestry by-products in creating value-added materials for green chemistry and sustainable polymer science.

## 2. Results and Discussion

### 2.1. Alder Bark Diarylheptanoids and Their Derivatives as Potential Biomass-Based Polyols

**Figure 1:**
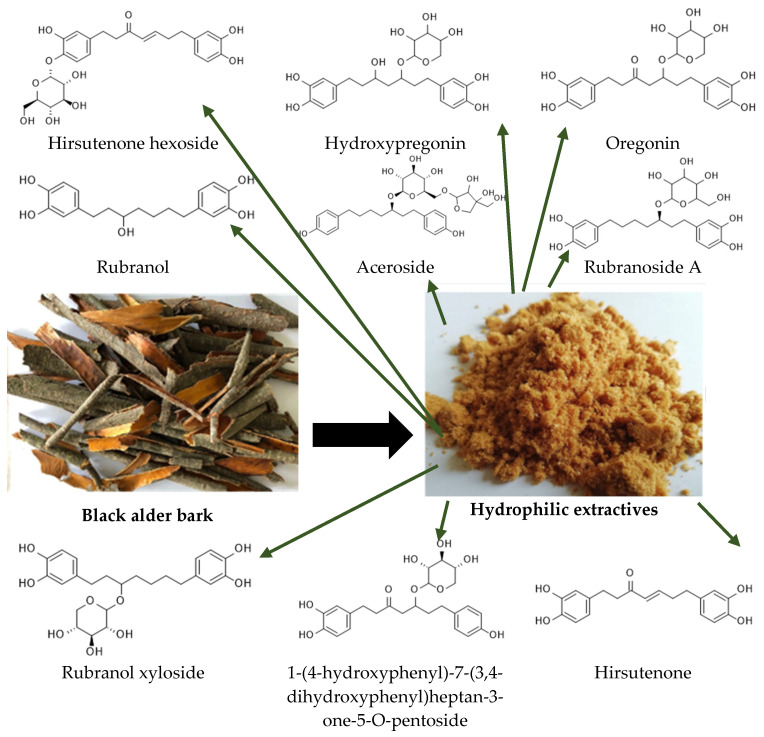
Chemical structures of diarylheptanoids and their derivatives isolated from black alder (*Alnus glutinosa*) bark.

The structures of the isolated diarylheptanoid compounds from black alder bark, predominantly containing sugar moieties such as the dominant oregonin, are shown in Figure 1.

**Figure 2:**
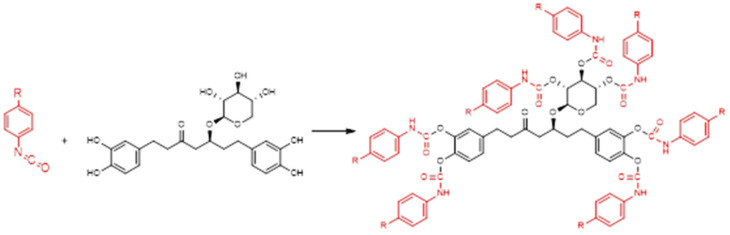
A schematic representation of the hypothetical structure of urethane bonds formed through the complete condensation of oregonin with isocyanates.

Firstly, these compounds were studied as the potential building blocks forming a PU matrix capable of reacting with isocyanate as polyols according to the scheme presented in Figure 2.

#### Obtaining of Model Diarylheptanoids for Introduction into PU Films

**Figure 3:**
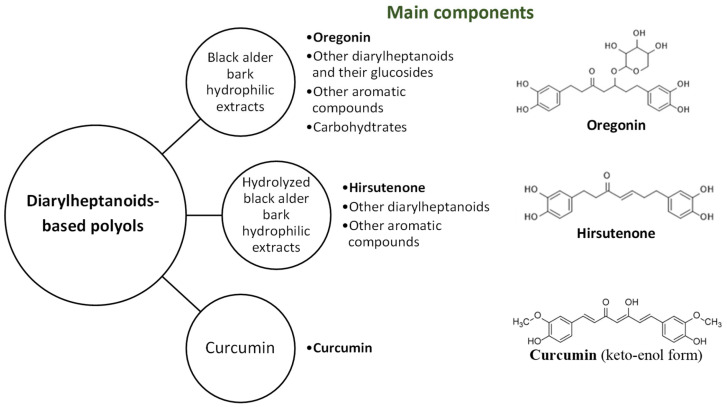
Diarylheptanoid-based polyols under study and their main constituents.

To characterize the potential of plant-derived diarylheptanoids and their derivatives of various structures, namely to reveal the influences of sugar moieties and various aromatic moieties, hydrolysis and fractionation techniques were used to isolate sugar-free diarylheptanoids from black alder bark extractives, which were then compared to unmodified extracts and the commercial diarylheptanoid curcumin (Figure 3).

Acid-catalyzed hydrolysis was used to remove the sugar moieties from the diarylheptanoid derivatives. The resulting sugar-free diarylheptanoids were then dissolved in tetrahydrofuran (THF), which is the solvent used for the synthesis of polyurethane films by casting. The carbohydrates obtained after hydrolysis are insoluble in THF and were therefore eliminated from the biomass-based polyol mixture. The obtained extracts were characterized in terms of yield, the yield of the THF-soluble fraction (sugar-free hydrolysate), the oregonin content, and overall composition using UPLC-Tof-MS. The parent extract contains 71.4% oregonin, which during hydrolysis is converted into a sugar-moiety-free oregonin analogue and hirsutenone. Oregonin is fully hydrolyzed after 3 h. Prolonged hydrolysis leads to the degradation of the hydrolysis products, both hirsutenone and the xylose-free oregonin analogue, which is most likely hydroxy-hirsutenone (Table 1).

**Figure 4:**
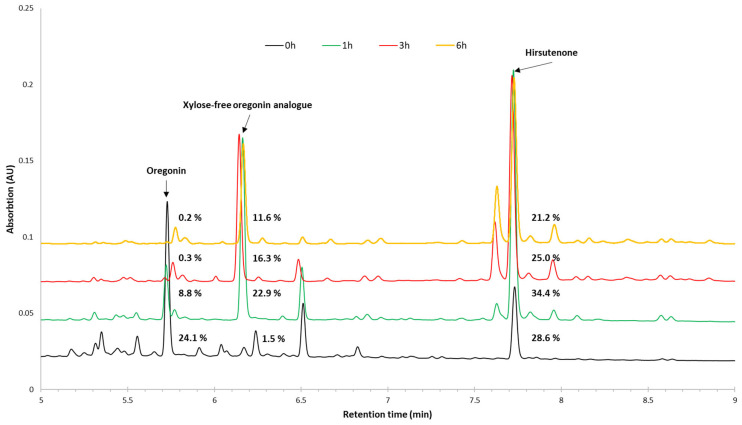
UPLC-UV chromatograms of black alder (*Alnus glutinosa*) bark extractives isolated by water and hydrolyzed using acid catalyst depending on hydrolysis time.

The UPLC-ToF-MS chromatograms representing the composition of the diarylheptanoids and their derivatives, as well as other constituents of the parent black alder bark extractives isolated by water and the hydrolyzed extracts depending on the hydrolysis time are shown in Figure 4.

Taking into account the highest yield of the sugar-free hydrolysate obtained after 1 h of hydrolysis (Table 1), as well as the highest content of hirsutenone and the sugar-free oregonin analogue in this product, 1 h hydrolysis is the optimal time for the highest concentration of aromatic compounds and the removal of carbohydrates and their derivatives.

### 2.2. The Effect of Various Diarylheptanoid-Based Building Blocks on the Properties of PU Films

#### 2.2.1. The Effect of Various Diarylheptanoid-Based Building Blocks on the Physical–Mechanical Properties of PU Films

The hydrolyzed (for 1 h) and non-hydrolyzed black alder bark extracts, as well as curcumin, were introduced into the PU films in a reaction with isocyanate at an NCO/OH ratio of one in the presence of the 1,4-diazabicyclo[2.2.2]octane catalyst. The extent of the substitution of commercial petrol-based polyol polyethylene glycol with an average molecular weight of 400 g/mol (PEG400) with biomass-based polyol was 81%, 64%, and 52% for the initial oregonin-enriched extract, the hydrolyzed hirsutenone-enriched extract, and curcumin, respectively, amounting to 30% of the biomass in PU. The effect of oregonin as a natural, highly functional biomass-based polyol on the properties of PU elastomers was the focus of our earlier research [8]. It was determined that incorporating oregonin at concentrations greater than 30% led to a high crosslink density in the PU matrix, which was accompanied by a drastic increase in the material’s friability. Therefore, in this study, the properties of PU elastomers synthesized from oregonin-, hirsutenone-, and curcumin-based polyols were compared at a biomass content of 30%. The reference PU films were synthesized under the same conditions using 100% PEG400 as polyol. The effects of diarylheptanoid-based polyols, on the mechanical properties of model PU films are summarized in Table 2. The OH functionality of the commercial polyol PEG400 is two. Based on the structures of oregonin and hirsutenone, their theoretical OH functionality values as polyols are seven and four, respectively. The chemical structure of curcumin contains a β-diketone group, which is prone to undergo keto-enol tautomerism. Based on the structure of the keto-enol form of curcumin (Figure 3), as well as the literature data suggesting that curcumin contains three hydroxyl functional groups that can react with isocyanates [23], the OH functionality of curcumin was considered to be three.

The introduction of diarylheptanoid-based extracts as polyols in the PU film matrix increases tensile stress—both maximal (σmax) and at break (σb), defined as strength—as well as the tensile modulus (E), thereby reducing the films’ flexibility (εb). The presence of sugar moieties in the structure of diarylheptanoids positively influences their mechanical strength (e.g., oregonin- vs. hirsutenone-based films). PU films containing diarylheptanoid curcumin are more flexible compared to hirsutenone-based films. It is known that the glass transition temperature (Tg) of polymers characterizes the segmental mobility of polymeric chains, which is restricted by crosslinking [35,36]. The increase in Tg in the order PEG 400-based film < curcumin-containing film < hirsutenone-containing film clearly indicates the increasing crosslinking density of the PU due to the increasing OH functionality of polyols in this order. It should be noted that the Tg of oregonin-based PU was not identified. This can be explained by the very low heat mobility of the PU matrix due to the highest functionality of oregonin. As a result, the thermal degradation of the material began without the transition from a glassy to a highly elastic state.

#### 2.2.2. The Effect of Various Diarylheptanoid-Based Building Blocks on the Biodegradation Behavior of PU Films

**Figure 5:**
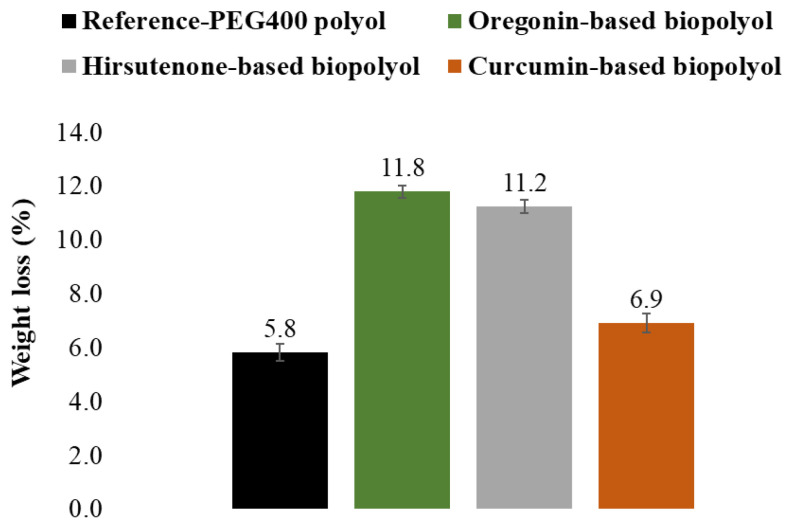
Weight loss of PU films depending on their composition after 60 days of biodegradation in compost-enriched soil. Extent of substitution of PEG400 with biomass-based polyol was 81%, 64%, and 52% for oregonin-based polyol, hirsutenone-based polyol, and curcumin, respectively, amounting to 30% of biomass in PU.

The synthesized PU films containing 30% by weight of various diarylheptanoid-based polyols, replacing PEG 400, were subjected to a degradation period of 2 months in compost-enriched soil, serving as both the medium and the source of biodegrading microorganisms. The susceptibility to biodegradation of the PUs under study was evaluated by their weight loss, as well as using FTIR and Py-GC/MS/FID techniques. After the 2-month biodegradation period in soil, the PU synthesized using conventional fossil-based building blocks experienced a weight loss of 5.8%. However, with the replacement of PEG 400 by bark polyol, the weight loss of the PU after biodegradation increased to 11.8% for the parent bark extract and 11.2% for the hydrolyzed bark extract. The weight loss of the PU synthesized using curcumin as the polyol was 6.9% (Figure 5). Cellulose, used as a reference material, experienced 100% weight loss during the 60 days of biodegradation under the same conditions.

Py-GC-MS/FID analysis allowed for the comprehensive examination of the changes in the PU materials after biodegradation. This was achieved by subjecting the sample to thermal degradation, resulting in the generation of a complex mixture of volatile products. The compounds associated with the urethane linkage were classified as N-containing compounds, such as aniline, diaminodiphenylmethane, and 1-isocyanato-4-methyl-benzene. The increase in the content of N-containing compounds in the pyrolysis products indicates the highest stability of the urethane linkage compared to those of the other linkages and functional groups within the PU network formed using PEG 400 or bark-sourced diarylheptanoids as polyols, resulting in their concentration after the degradation of other PU building blocks (Table 3).

The decrease in the content of aromatic compounds in the pyrolysis products after the biodegradation of the bark diarylheptanoid-based PUs show that alder bark-sourced diarylheptanoids are susceptible to biodegradation by soil microorganisms. In contrast, the aromatic moieties introduced by curcumin into the PU matrix are not biodegradable, as evidenced by the concentration of aromatic pyrolysis products in the PU material after biodegradation (Table 3). This can be explained by the significant role of the catechol moiety in the structure of biomass-based polyols of diarylheptanoid nature, which promotes the biodegradation of PU materials based on these biomass-based polyols.

**Figure 6:**
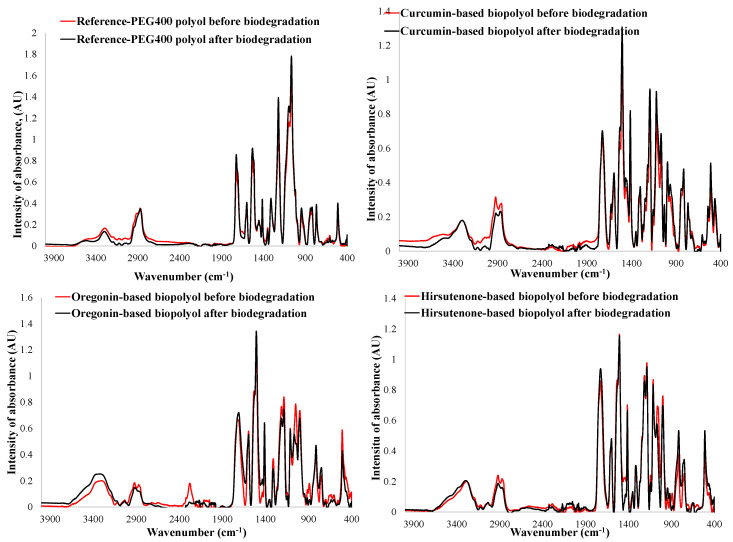
FTIR spectra of PU films with different formulations before and after 60 days of biodegradation in compost-enriched soil. Extent of substitution of PEG400 with biomass-based polyol was 81%, 64%, and 52% for oregonin-based polyol, hirsutenone-based polyol, and curcumin, respectively, amounting to 30% of biomass in PU.

FTIR spectroscopy confirmed that the substitution of PEG400 with diarylheptanoids as building blocks significantly enhances the susceptibility of PU materials to biodegrade in soil. This is indicated by more prominent chemical changes in their structure, specially decreased aliphatic C-H signals (2925–2869 cm^−1^). Additionally, there is an increase in the N–H and O-H stretching signals (3321–3296 cm^−1^) of the oregonin-based PUs (Figure 6). The later augmentation in the PU samples is commonly linked to the hydrolysis of ether bonds, or both ethers and urethane bonds. Since no increase in the intensity of FTIR peaks corresponding to O-H and N-H stretching was observed after the biodegradation of the polyether PU synthesized solely using PEG400 as a polyol, as well as using sugar-moiety-free diarylheptanoids as a curcumin and hirsutenone-based mixture, it can be concluded that the hydrolysis of glucosidic bonds in oregonin and similar sugar-containing diarylheptanoid derivatives integrated into the PU matrix occurs.

### 2.3. The Effect of Oregonin-Based Building Blocks on the Properties of PUR Foams

**Figure 7:**
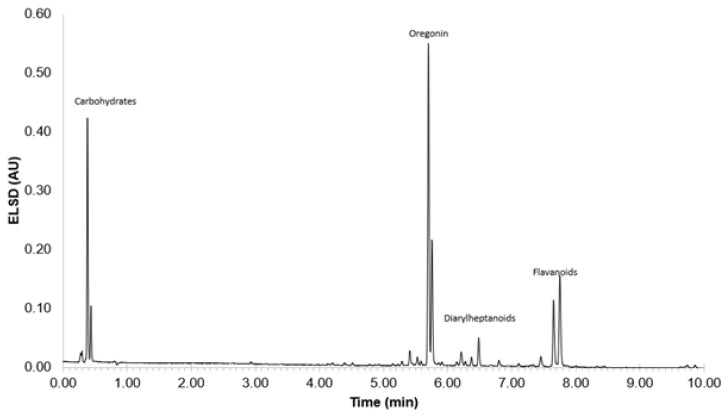
UHPLC-ELSD chromatogram of black alder (*Alnus glutinosa*) bark extractives used for PUR foam synthesis.

Based on the studies conducted with PU films, black alder bark extracts enriched with both sugar and diarylheptanoid moieties containing oregonin as the main component are recognized as the most useful biomass-based polyols for the synthesis of PU materials. Therefore, the oregonin- and carbohydrate-based composition obtained via water extraction at 90 °C using microwave-assisted extraction was selected for subsequent PUR foam synthesis. UHPLC-ELSD experiments were used to monitor the composition of a mixture of isolated extractives (Figure 7).

**Figure 8:**
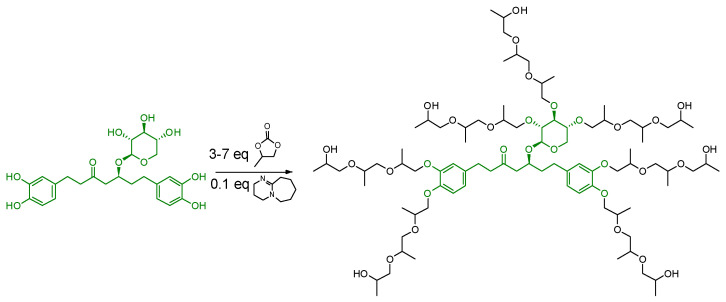
Reaction scheme for oxyalkylation of oregonin with propylene carbonate in presence of catalyst.

For the synthesis of the PU films, the mixture of black alder bark extractives isolated by water was simply fractionated using THF as a solvent, and the undissolved compounds were removed. The introduction of diarylheptanoid-based extractives in the matrix of PUR foams is more challenging as polyurethane foams are made into a solvent-free medium on the one hand and require the polyol component to be into a liquid form on the other hand. Therefore, two approaches to the liquification of oregonin-enriched alder bark extractives were developed as described in [10,15]. One of the approaches involved using oxypropylated glycerol “Lupranol 3300”, transforming the bark extractives into a liquid form. The partial oxidation of catechol units and the transformation of carbohydrates into organic acids was observed during the liquification process. The liquefied biomass contained aliphatic, various phenolic, and carboxyl OH groups. The second approach involved the innovative “green’’ modification of the extractives with propylene carbonate in the presence of 1,8-diazabicyclo [5.4.0] undec-7-ene as a catalyst, producing liquid biomass-based polyols with uniform functionality, characterized by the presence of solely aliphatic OH groups (Figure 8).

The black alder bark extractives liquefied using Lupranol 3300 and the oxypropylated black alder bark extractives, hereafter referred to as liquefied oregonin-based polyol and oxypropylated oregonin-based polyol, were utilized for the synthesis of PUR foams. Their hydroxyl value (OHV), acid value, water content, and viscosity are presented in Table 4.

#### 2.3.1. The Reinforcing Effect of Oregonin-Based Building Blocks on the PUR Foams

Similar to the PU films, the PUR foams containing oregonin-derivatives as 100% building blocks replacing commercial polyol exhibited significantly better compression characteristics than those of the reference composition utilizing only commercial fossil-derived building blocks (Table 5).

The most reinforcing effect was observed for the foam synthesized using polyol on the basis of oxypropylated oregonin-enriched extracts. This can be explained by two factors: the high functionality of polyols and the availability of their OH groups, which are freed from electronic and steric constraints as a result of oxypropylation, allowing for interaction with isocyanate.

Considering that an increase in OH functionality does not always enhance all properties, and in some cases, may lead to undesirable effects, such as increased brittleness, alterations in cell structure, and challenges in processability, we have previously optimized the composition of polyurethane foams by controlling the average OH functionality of biomass-based polyols using both the methods of oregonin-based polyol synthesis—oxypropylation and liquefaction [11]. In the oxypropylation process, adjusting the ratio of propylene carbonate to oregonin regulates the content of bifunctional propylene diols in the final biomass-based polyol. Similarly, in the liquefaction method, modifying the oregonin-to-liquefying agent ratio enables the synthesis of biomass-based polyols with tailored functionality and OH content. By optimizing these parameters, we increased OH functionality to its optimal level without exceeding it, thereby avoiding undesirable effects.

#### 2.3.2. The Effect of Oregonin-Based Building Blocks on the Biodegradation Behavior of PUR Foams

**Figure 9:**
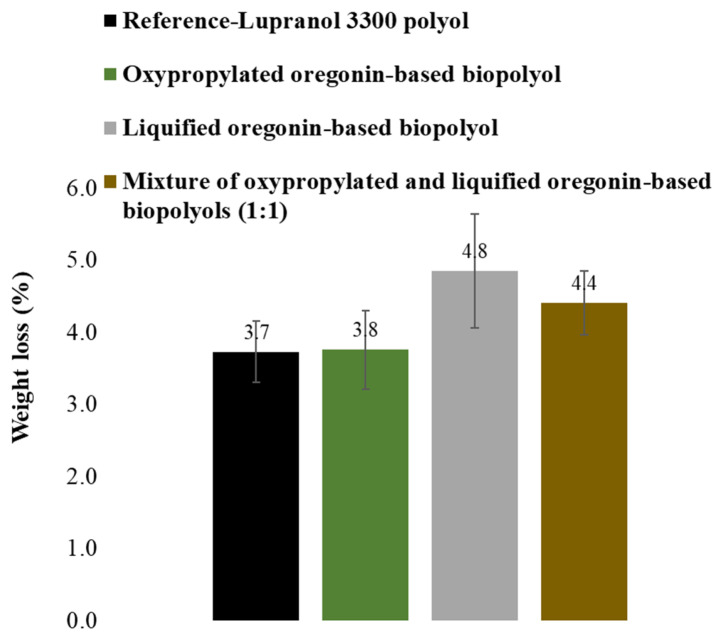
Weight loss of PUR foams depending on their composition after 60 days of biodegradation in compost-enriched soil.

Despite the previous findings suggesting that incorporating oregonin-based polyols into the composition of PU films enhances their biodegradability (see Section 2.2.2), the results of the biodegradation tests on the synthesized PUR foams indicate a different behavior. The PUR foams synthesized using oxypropylated oregonin-based polyol and liquified oregonin-based polyol exhibited resistance to biodegradation similar to the PUR foams synthesized using commercial polyol Lupranol 3300. This was demonstrated by a maximum weight loss of only about 5% for the oregonin-based PUR foams after a 2-month biodegradation test under composting conditions compared to about 4% weight loss for the reference PUR composition based on the commercial polyether polyol Lupranol 3300 (Figure 9).

These findings suggest that bark-based PUR foams are well suited for applications such as insulation in environmentally exposed settings. Such a difference in the biodegradation behaviors of oregonin-based PU films and foams can be explained by the disappearance of catechol groups of oregonin and other diarylheptanoids during the liquification of alder bark extractives according to the requirements of PUR foams synthesis, which we previously in this work admitted to being crucial for susceptibility to biodegradation.

### 2.4. The Effect of Oregonin-Based Building Blocks and Additives on the Thermo-Oxidative Resistance of PU Materials

#### 2.4.1. The Effect of Oregonin-Based Building Blocks on the Thermal Resistance of PU Films and Foams

**Figure 10:**
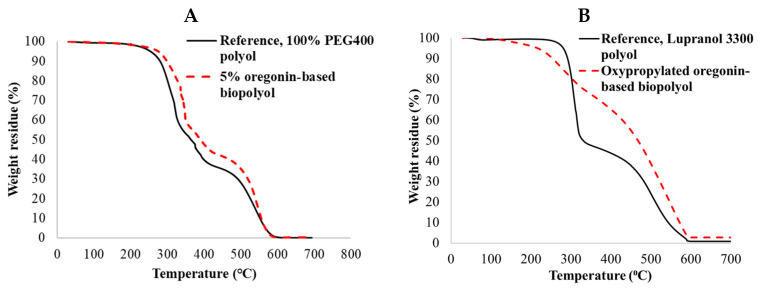
TG curves of PU films (**A**) and foams (**B**) synthesized using reference commercial polyols and oregonin-based polyols in air.

Recognizing the presence of hindered phenolic OH groups in the structure of oregonin, the antioxidative effect of oregonin-based polyols was also tested in the PU films and foams. The introduction of a low content (5%) of oregonin-based extractives into the composition of the PU films increased their thermo-oxidative resistance (Figure 10A). Oregonin-based PUR foams also demonstrated enhanced thermal resistance, as evidenced by reduced material weight loss during the volatile formation stage (200–350 °C) and increased weight retention at higher temperatures (450–600 °C), primarily associated with char combustion (Figure 10B).

#### 2.4.2. The Effect of Oregonin-Based Building Blocks on the Combustion Characteristics of PUR Foams

**Figure 11:**
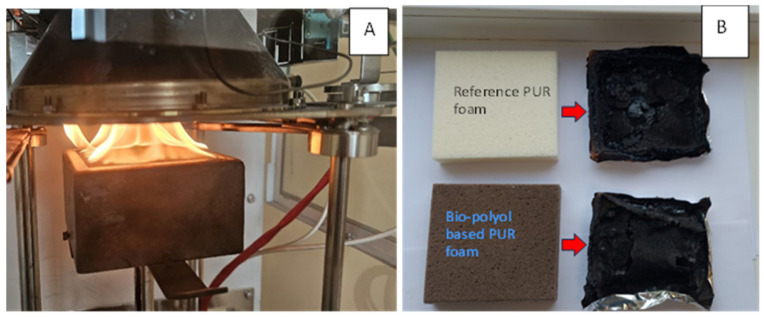
The cone calorimeter tests procedure (**A**). The reference and biomass-based polyol-derived PUR foam samples before and after the cone calorimeter test (**B**).

It is known that the flammability of PUR materials is linked to the release of flammable gaseous decomposition products during the initial degradation stage [30]. Therefore, the biomass-based polyol-derived PUR foam is expected to be less flammable compared to the reference. To quantitatively evaluate material flammability, the combustion tests were performed using the cone calorimeter method ISO 5660-1:2015. The fire-related behaviors of the PUR foams produced from a 7:3 mixture of two commercial polyols, Lupranol 3300 and Lupranol 3422 (reference sample), were compared to those of the foams synthesized from a 1:1 blend of two biomass-based polyols derived through the acid-free liquefaction and ‘green’ oxypropylation of black alder bark extractives utilizing the commercial polyether polyol Lupranol 3300 and propylene carbonate, respectively (Figure 11).

The cone calorimeter test is based on the principle of oxygen consumption, which is widely used for the quantitative evaluation of material flammability. During the test, oxygen consumption and weight loss are the primary parameters measured experimentally by a device. Additionally, smoke yield is quantified using a laser detector. Other characteristics are automatically calculated using the cone calorimeter’s software (Fire Testing Technology ConeCalc software). A wide range of data determining the behavior of PUR combustion during combustion were measured, including the time of ignition (TTI), the time of flameout (TTF), mass losses (Δm), total heat release (THR), the average (Av-HRR) and peak heat release rates (PHRR), the average effective rate of combustion (Av-HER), total smoke release (TSR), the maximum average rate of heat emission (MARHE), and the average yields of CO_2_ and CO [37] (Table 6).

**Figure 12:**
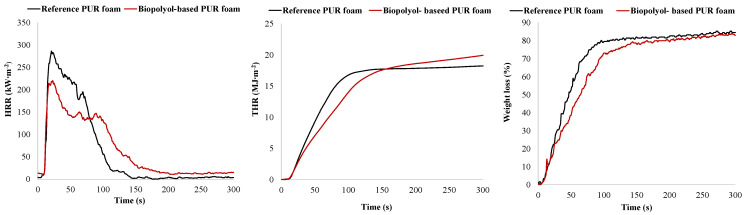
Heat release rate (HRR), total heat release (THR), and weight loss of PUR foam samples as function of combustion duration measured using cone calorimetric tests.

Mass loss (Δm) during the combustion test ranged from 77.2% to 79.6%, which is considered consistent when accounting for deviations in the repeated test results. The ignition time of the reference PUR foam was slightly longer compared to that of the bio-polyol-based PUR foam. However, the time to flameout for the bio-polyol-based PUR foam was 30% longer than that of the reference sample. This extended flameout time was accompanied by a lower heat release rate (HRR) at the initial stage of combustion, and consequently a more prolonged combustion period. As a result, the PHRR for the bio-polyol-based foam was 227 kW∙m^−2^ compared to 276 kW∙m^−2^ for the reference foam. The total heat released (THR) during the first 150 s of combustion was also lower for the bio-polyol-based foam. However, between 150 and 300 s, the THR for the reference sample decreased relative to the bio-polyol-based PUR foam. At the end of the test, the THR and EHC values for the reference PUR foam were 12–13% lower than those for the bio-polyol-based foam (Table 6). THR reflects the total heat released during combustion, including both gaseous products and char residue, while the EHC describes the extent of combustion of volatile products generated during the pyrolysis of PUR foam [37]. The HRR and THR data are further supported by the weight loss dynamics of the foam samples, which indicate the faster degradation of the reference samples (Table 6, Figure 12).

For example, during the first 50% of sample weight loss, the average specific mass loss rate (Av-MLR (mA, 0–50)) of the biomass-based PUR foam was 40% lower compared to that of the reference foam. Over the range from 10% to 75% weight loss, the average specific mass loss rates (Av-MLR (mA, 10–75)) for the reference and biomass-based polyol-derived PUR foam samples were 13.4 g∙(s∙m^2^)^−1^ and 9.0 g∙(s∙m^2^)^−1^, respectively (Table 6).

**Figure 13:**
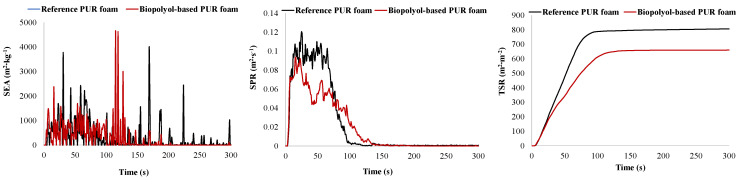
Smoke emission area (SEA), smoke production rate (SPR), and total smoke released (TSR) depending on combustion duration using cone calorimetric tests.

It was observed that the higher degradation rate of the reference material was accompanied by a significantly greater content of non-flammable by-products such as smoke in the combustion products of the reference PUR foam compared to that of the biomass-based polyol derived foam (Table 6; Figure 13).

The total smoke released (TSR) during the combustion tests on the reference PUR foam was approximately 25% higher than that of the bio-polyol-based PUR foam. Consequently, a larger portion of carbon was emitted as particulate matter along with gaseous products during the pyrolysis of the reference PUR foam. Unlike the volatile products of PUR degradation, these particulate matters were not oxidized, remaining as solid air pollutants.

In contrast, the bio-polyol-based PUR foam exhibited the more complete combustion of its decomposition products, resulting in higher values of HRR, THR, and CO_2_ yield. This trend became dominant after 150 s of combustion, when the degradation rates for both the samples were similar. However, during the first 150 s of combustion, the lower degradation rate of the bio-polyol-based foam was the key factor responsible for reduced heat release compared to that of the reference material, despite the higher smoke content in the pyrolysis products of the reference foam.

Based on the dependence of THR, HRR, and weight loss on combustion duration, the bio-polyol-based PUR foam can be characterized as less flammable compared to the reference PUR foam. Additionally, the maximum average heat release rate (MARHE) of the bio-polyol-based foam was 142 kW/m^2^, which is significantly lower than the 191 kW/m^2^ observed for the reference sample, further demonstrating its reduced tendency for fire development [37]. The average carbon monoxide yields for the reference and biomass-based polyol-derived PUR foams were similar taking into account the standard deviation values (Table 5).

#### 2.4.3. The Effect of Oregonin-Based Additive on the Color Change in Commercial Rigid Polyurethane Foam System Elastopir 1132/509/0 According to the Results on Thermal Oxidative Aging

The effect of the oregonin-based black alder bark extractives as antioxidative additives was tested in the commercial rigid polyurethane foam system Elastopir 1132/509/0. Elastopir is a rigid polyurethane (PUR) foam system based on polyisocyanurate developed by BASF which is widely used in the building industry as a heat isolation material with effective fire protection [38]. The main areas of application are pitched and flat roof insulation, as well as cavity wall and floor insulation. The anti-aging effect of the oregonin-based extractives was compared with that of commercial radical scavenger Irganox 1010 commonly used in PU materials [39].

**Figure 14:**
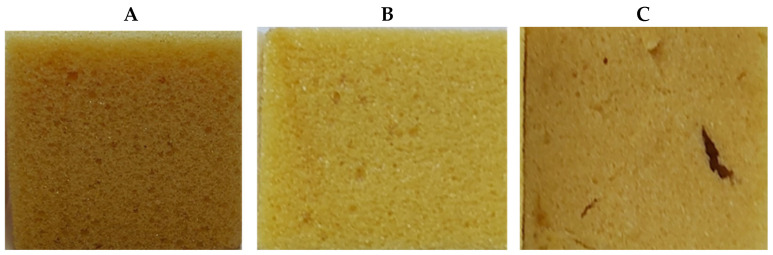
PUR foam samples after thermal oxidative aging at 150 °C for 24 h: reference (**A**), and containing 1% of oregonin-based black alder (*Alnus glutinosa*) bark extractives (**B**) and 1% of Irganox (**C**).

The PUR foam color darkened due to thermal oxidative aging, which was observed for all the compositions. The extent of browning was most pronounced in the reference systems. The addition of both the extractives and Irganox reduced the extent of darkening compared to that of the reference composition (Figure 14).

**Figure 15:**
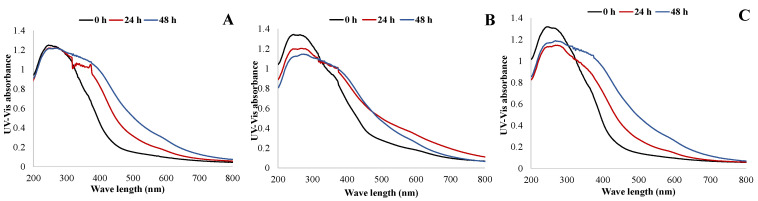
UV-visible solid-phase spectra before (0 h) and after thermal oxidative aging at 150 °C for 24 and 48 h for different PUR foams: reference (**A**), oregonin-based black alder (*Alnus glutinosa*) bark extractives (**B**) and Irganox (**C**) as antioxidants.

An increase in PUR foam discoloration due to thermal oxidative aging was observed for all the compositions as the duration of thermal oxidative stress increased. This was accompanied by more intense absorbance on the material’s surface across most of the UV-visible range (Figure 15).

**Figure 16:**
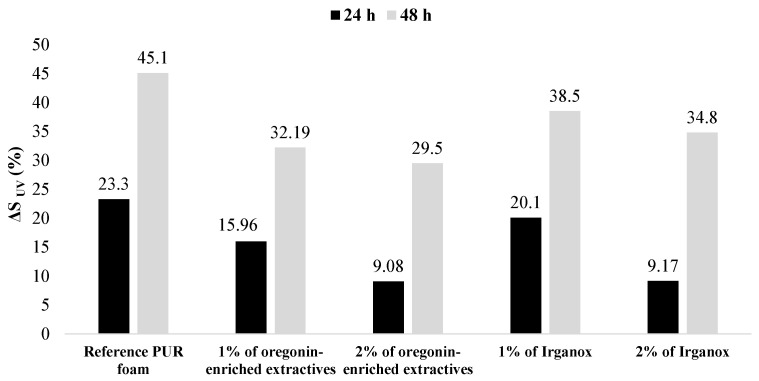
Increment in area under UV-visible absorbance curves for PUR foams surface after thermal oxidative aging for 24 and 48 h.

The increment in area under the UV-visible absorbance curves (ΔS values) calculated for initial PUR foam composition and those containing oregonin-based black alder bark extractives and commercial antioxidant Irganox is presented below (Figure 16). The extent of discoloration was most pronounced in the reference systems. The addition of both the extractives and Irganox reduced the extent of discoloration compared to that of the reference composition (Figure 16).

Importantly, the antioxidative effect of the oregonin-enriched extractives was more significant than that of the commercial antioxidant Irganox. At a concentration of 1% by weight, the antioxidative effect of the oregonin-based black alder bark extractives was more pronounced at both 24 and 48 h of aging. A similar effect was observed at a 2% by weight concentration after 24 h of aging. However, after 48 h, the diarylheptanoid-based extractives demonstrated a more significant effect. Obviously, due to the lower reactivity of the phenolic groups in the catechol units of oregonin with isocyanates compared to that of aliphatic OH groups, the free phenolic groups that did not condense with the isocyanates present in the cured PU matrix. Their concentration is sufficient for oregonin to function both as a crosslinking component and a radical scavenger simultaneously.

## 3. Materials and Methods

### 3.1. Materials

#### 3.1.1. Materials for Obtaining Diarylheptanoid-Based Polyols

Black alder (*Alnus glutinosa*) bark, rich in diarylheptanoid glucosides as secondary metabolites, was harvested from approximately 27-year-old trees grown in the Talsi municipality of Latvia. Deionized water was used for the microwave-assisted hot water extraction of diarylheptanoid-enriched extractives from the black alder bark. Tetrahydrofuran (THF) with a purity of ≥99.9% was used to fractionate the obtained extractives, making the THF soluble fraction suitable as biomass-based polyols for the synthesis of polyurethane films.

Sulfuric acid (purity ≥ 95%), ammonia (≥35%), and anhydrous THF (purity ≥ 99.9%) purchased from Merck (Darmstadt, Germany) were used for the hydrolysis and fractionation of the alder bark extractives to obtain sugar-free diarylheptanoids.

The commercial oxypropylated glycerol-based polyol-polyether Lupranol 3300 (OHV = 400 mg KOH·g^−1^) from BASF (Ludwigshafen, Germany) and propylene carbonate (PC) from Sigma-Aldrich (Waltham, MA, USA), along with the oxypropylation catalyst 1,8-diazabicyclo[5.4.0]undec-7-ene (DBU, purity ≥ 99%), were used as liquefaction and oxypropylation agents. These agents were employed to make the diarylheptanoid-enriched extractives from the black alder bark suitable as biomass-based polyols for the synthesis of rigid polyurethane foams.

#### 3.1.2. Materials for Obtaining Polyurethane Films and Foams

In addition to the THF-soluble diarylheptanoid-enriched fraction of the black alder bark extractives used as biomass-based polyols, curcumin (purity ≥ 75%), and commercial petroleum-based polyol polyethylene glycol with an average molecular weight of 400 g/mol (PEG400, purity ≥ 95%), commercial polymeric diphenylmethane diisocyanate (PMDI) with [NCO] = 7.5 mmol·g^−1^ and an average functionality of 2.7, as well as the catalyst 1,4-diazabicyclo[2.2.2]octane (DABCO) were purchased from Merck (Germany) for use in the synthesis of polyurethane (PU) films. Anhydrous tetrahydrofuran (THF, purity ≥ 99.9%) from Merck was used as the reaction medium for PU film synthesis.

For the creation of biomass-based and reference polyurethane (PUR) foam compositions, the liquid biomass-based polyols obtained by the modification of diarylheptanoid-enriched extractives, commercial oxypropylated glycerol-based polyol-polyether Lupranol 3300 (OHV = 400 mg KOH·g^−1^; acid value < 5 mg KOH·g^−1^) from BASF (Ludwigshafen, Germany), six functional commercial samples of oxypropylated sorbitol-based polyol-polyether Lupranol 3422 (OHV = 490 mgKOH∙g^−1^; acid value < 5 mg KOH·g^−1^) from BASF (Ludwigshafen, Germany), and commercial polymeric diphenylmethane diisocyanate (PMDI) with [NCO] = 7.5 mmol·g^−1^ and an average functionality of 2.7 from BASF were used. Other components included the amine catalyst Polycat 5 (Evonik, Essen, Germany), the physical blowing agent Opteon™ 1100 (Chemours, Wilmington, DE, USA), and the surfactant Niax Silicone L-6915 (Momentive Performance Materials Inc., Leverkusen, Germany).

The oregonin-enriched fraction isolated from the black alder bark extractives was tested as a natural antioxidant to reduce the thermal oxidative aging of the commercial polyurethane foam system Elastopir 1132/509/0.

The commercial radical scavenger Irganox 1010 from BASF (Ludwigshafen, Germany) was used as the reference antioxidant.

### 3.2. Isolation and Characterization of Diarylheptanoid Derivatives

Hydrophilic diarylheptanoid glycosides were extracted from the black alder (*Alnus glutinosa*) bark using microwave-assisted extraction as described in [14]. Briefly, finely milled bark was mixed with deionized water at a 1:5 (*w*/*w*) ratio to form a suspension, which was then subjected to dielectric heating in a microwave-assisted extraction system. The mixture was heated to 90 °C within approximately 3 min. Upon reaching the target temperature, a rapid pressure drop was applied to disrupt the plant cell walls, thereby enhancing the release of diarylheptanoid compounds into the liquid phase. The resulting extract was separated from the solid residue by vacuum filtration using a Buchner funnel, and subsequently lyophilized.

The obtained extract enriched with diarylheptanoid glycosides was modified to create a model mixture of sugar-free diarylheptanoids using hydrolysis and fractionation techniques. Approximately 300 mg of the extract was weighed into pressure tubes, and 3.5 mL of deionized water was added. The mixture was vortexed until fully dissolved. Next, 125 µL of 72% sulfuric acid was added, and the samples were incubated in a thermostat set to 105 °C. The samples were removed after 0, 1, 3, and 6 h, and 350 µL of ammonia was added to each sample to neutralize the reaction. The samples were then freeze-dried. Subsequently, 10 mL of anhydrous THF was added to each sample, the solutions were stirred for 24 h, and then filtered to remove undissolved carbohydrates. The solvent was evaporated from the THF solution using a rotary evaporator. The resulting fraction was redissolved in water and dried via lyophilization to produce a mixture of sugar-free diarylheptanoids.

The liquid chromatography analysis of the isolated diarylheptanoids and their derivatives was conducted as described in [16].

### 3.3. Introduction of Diarylheptanoids as Building Blocks into PU Films

To prepare biomass-based polyols for the synthesis of polyurethane (PU) films, all the diarylheptanoid-enriched extracts were dissolved in anhydrous THF. The soluble fraction of the extracts was isolated and utilized as biomass-based polyol in the synthesis of PU films. Specified amounts of each component of the PU system, including the diarylheptanoid-based polyols, the commercial polyol PEG 400, DABCO, and PMDI, were separately dissolved in extra-dry THF at 20 °C. These components were then combined and pre-polymerized in the THF solution for 20 min under vigorous mixing, followed by a 2 min treatment in an ultrasonic bath. Subsequently, 45 g of the solution containing approximately 10.0 g of the pre-polymer was poured into a Teflon mold and covered with a flat glass plate. After several hours, the glass cover was removed, allowing the solvent to evaporate slowly overnight. The resulting films were then transferred to a desiccator containing P₂O₅ for further curing at 25 °C over 5 days. This was followed by post-curing in an oven at 90 °C for 8 h. The biomass-based polyol content in the PU system was set at 30%, with an NCO/OH ratio of 1.0.

### 3.4. Introduction of Diarylheptanoids as Building Blocks into PUR Foams

One approach for incorporating diarylheptanoid-based building blocks into polyurethane (PUR) foams in the form of liquid polyol involved using oxypropylated glycerol (Lupranol 3300) as a liquified agent and as a co-reagent simultaneously. A combination of processes, including solvolysis, the dissolution of extractives, and their modification via partial condensation with OH groups of Lupranol 3300, transforms the extractives into liquid polyols. This was achieved by liquefying 300 g of 30% suspension of diarylheptanoid-enriched extractives in Lupranol 3300. The suspension was placed in a round-bottom flask equipped with a magnetic stirrer and heated in a silicone oil bath with automatic temperature control. The process was conducted at 150 °C under a constant flow of argon for 6 h. The insoluble fraction was subsequently removed by filtration through a glass filter (pore size = 3 µm) preheated to 120 °C. The obtained liquified diarylheptanoid-based polyol contained aliphatic, phenolic, and carboxylic hydroxyl (OH) groups and was used for the synthesis of PUR foams [15].

Another approach for incorporating diarylheptanoid-based building blocks into polyurethane (PUR) foams involved modifying isolated black alder diarylheptanoids and their derivatives through a reaction with propylene carbonate (PC). A mixture comprising 100 g of diarylheptanoid-enriched extractives, 456 g of PC, and 17 g of 8-diazabicyclo[5.4.0]undec-7-ene (DBU) as the catalyst was prepared in a 1 L two-neck round-bottom flask equipped with a mechanical stirrer and a reflux condenser. The flask was placed in a silicone oil bath with precise temperature control, and the reaction was carried out at 150°C under constant stirring and in an argon atmosphere for 24 h. This process yielded 373 g of biomass-based polyol, corresponding to an approximate 65% yield based on the weight of the initial suspension. The resulting product was used for the synthesis of PUR foams as a highly reactive liquid biomass-based polyol with uniform functionality, characterized by the presence of aliphatic hydroxyl (OH) groups [15].

Polyurethane (PUR) foams were produced using the free-rise method with a two-component system consisting of the previously described diarylheptanoid-based polyols and isocyanate. The commercial polyol Lupranol 3300, or a 7:3 mixture of Lupranol 3300 and Lupranol 3422, was used as the reference. The weight of PMDI required was calculated using Equation (1):

(1) $$M_{pMDI}=\left(\frac{M_{1OH}}{{EW}_{1}}+\frac{M_{2OH}}{{EW}_{2}}+\frac{{2M}_{H2O}}{18}\right)\times 133.3\times 1.15$$

The notations are listed as follows:

M_pMDI_—weight of pMDI (g);
M_1OH_—weight of polyol 1 (g);
M_2OH_—weight of polyol 2 (g);
M_H2O_—weight of water in system (g);
18-molar weight of water (g∙mol^−1^);
EW_1_—equivalent weight of polyol 1 (g∙Eq^−1^);
EW_2_—equivalent weight of polyol 2 (g∙Eq^−1^);
133.3—equivalent weight of pMDI (g∙Eq^−1^);
1.15—molar NCO/OH ratio.

To prepare the polyol system, 30 g of one or a combination of two biomass-based polyols, or the reference polyol (Lupranol 3300 or a 1:1 mixture of Lupranol 3300 and Lupranol 3422) was placed in a paperboard cup shaped like an inverted cone (d_1_ = 72 mm; d₂ = 97 mm; H = 180 mm). Specified amounts of amine catalyst (Polycat 5), surfactant (Niax Silicone), and water were added, and the mixture was thoroughly stirred at 2000 rpm for approximately 1 min using a high-speed mechanical stirrer. The blowing agent, Opteon^TM^ 1100, was then added as the final ingredient, and the polyol system was premixed again at 2000 rpm for 15–20 s. Subsequently, polymeric diphenylmethane diisocyanate was added in an amount sufficient to achieve an NCO/OH molar ratio of 1.15, and the mixture was stirred for 10 s. Foaming was conducted in the same cup. After 24 h of post-curing at room temperature, excess foam above the cup was trimmed. In all cases, the samples for PUR foam testing were taken from a 65 mm deep layer of material located below the top of the cup [15].

### 3.5. Characterization of PU Materials Containing Diarylheptanoid-Based Building Blocks

#### 3.5.1. Mechanical Properties of Polyurethane Films and Foams

The tensile properties of the polyurethane (PU) films were evaluated following the ASTM D882-12 standard using a ZWICK/Roell Z100 universal testing machine (Ulm, Germany). Testing was conducted under controlled conditions, with a temperature of 21 °C and a relative humidity of 60%. The sample dimensions were specified as follows: length of 100 mm, width of 5.0 mm, and thickness ranging from 0.200 mm to 0.250 mm. The crosshead separation distance was set at 70 mm. For each sample, six to eight specimens were tested to ensure reliability. The key mechanical properties, including Young’s modulus, tensile strength at break, and elongation at break, were calculated using ZWICK software (testxpert II).

The compressive strength and modulus of the PUR foams along the foaming direction were evaluated in accordance with the ISO 844:2021 standards. Testing was performed using a Zwick/Roell Z100 universal testing machine (Zwick Roell, Ulm, Germany) with a maximum test load of 1 kN and a deformation rate of 10% of the sample height per minute. For each PUR foam composition, six cubic samples with dimensions of 30 mm were tested.

The apparent density of PUR foams was determined following the ISO 845:2006 standard.

#### 3.5.2. Biodegradation Behavior of Polyurethane Films and Foams

Polyurethane biodegradation behavior was characterized using a soil burial test. Rectangular strips of the polyurethane (PU) films (approximately 1 cm × 7 cm) and cubic samples of the polyurethane (PUR) foams (1–1.5 cm) were prepared for various compositions. To ensure the absence of residual moisture, the specimens were meticulously dried before the experiment. Each dried sample was then weighed using a high-precision analytical scale (resolution: 0.0001 g). The soil burial test method for assessing polyurethane biodegradation was adapted from [40]. For this study, commercially available Biolan peat-based soil (Finland) was used. The soil was supplemented with magnesium-containing limestone powder (4 kg/m^3^) and had the following nutrient composition: nitrogen (1300 mg∙kg^−1^ dry matter), phosphorus (840 mg∙kg^−1^ dry matter), and potassium (4200 mg∙kg^−1^ dry matter). Additional soil properties included a pH of 6.5, electrical conductivity of 40 mS∙m^−1^, bulk density of 300 g∙L^−1^, moisture content of 60%, and fraction size <35 mm. To enhance microbial activity, 40 g∙kg^−1^ compost derived from the “Getiņi Eco Ltd.” (Riga, Latvia) landfill was added and thoroughly mixed into the soil. Each test included 0.1–0.7 g of prepared PU material combined with 50 g of soil/compost mixture in a 500 mL polypropylene jar. The jars were sealed with perforated caps to ensure oxygen availability. Each test was replicated five times. The jars were incubated in the dark at 21 ± 2 °C for 60 days. To maintain optimal moisture levels, 10 mL of tap water was added to each jar weekly. After 60 days incubation, the PU specimens were washed to remove soil residues and dried at 50 °C for 48 h. The dried samples were reweighed using the same analytical scale, and weight loss was calculated as a percentage of the initial weight.

The structural properties of the PU networks were assessed before and after biodegradation using FTIR spectroscopy and pyrolysis gas chromatography/mass spectrometry/flame ionization detection (Py-GC/MS/FID) analyses.

The PU films were analyzed using the attenuated total reflectance (ATR)–FTIR method, while the cryogenically ground PUR foams were studied using the KBr technique. FTIR analysis was conducted using a Spectrum One FTIR spectrometer (Perkin Elmer, Waltham, MA, USA) equipped with an ATR diamond–ZnSe top plate. The parameters included a spectral range of 4000–400 cm^−1^, a resolution of 4 cm^−1^, and 64 scans. Baseline correction was performed using Spectrum version 5.0 software, and spectra were normalized to the sum intensity of all peaks. Duplicate analyses were performed, and the averaged results were used for further evaluation.

The key parameters of Py-GC/MS/FID analysis were as follows: a mass of cryogeniccaly ground sample of 1.00–2.00 mg (moisture content < 1%), a pyrolysis temperature of 500 °C, and a heating rate of 600 °C∙s^−1^ Analysis utilized a Micro Double-shot Pyrolyzer Py-3030D (Frontier Laboratories, Ltd., Fukushima, Japan) coupled with Shimadzu GC/MS/FID-QP ULTRA 2010 (Kyoto, Japan) apparatus equipped with an RTX-1701 capillary column (60 m × 0.25 mm × 0.25 μm). The operational parameters included an injector temperature of 250 °C, an ion source energy of 70 eV (electron impact), a mass scan range of *m*/*z* 15–350, and helium as the carrier gas (1 mL∙min^−1^ flow rate; split ratio: 1:30). The oven program consisted of initial isothermal hold at 60 °C for 1 min, ramp at 6 °C∙min^−1^ to 270 °C, and final hold at 270 °C for 10 min. Compounds were identified with GC/MS chromatography using the NIST 11 and NIST 11 s libraries. The relative peak areas (% chromatogram) were calculated using Shimadzu software (LabSolutions DB 4.20) with GC/FID data. Four replicate pyrolysis experiments were conducted, and the results were averaged. The coefficient of variation for measurements was ≤10%.

#### 3.5.3. Non-Isothermal Thermogravimetric Analysis of Polyurethane Films and Foams

The cryogenically milled polyurethane (PU) film and polyurethane foam (PUR) samples were subjected to non-isothermal thermogravimetric analysis in an air atmosphere. Analysis was conducted using a Seteram Setline device (Seteram, Caluire-et-Cuire, France). Approximately 20 mg of each sample was placed in an alumina crucible for testing. The temperature range was programmed from 25 °C to 700 °C at a constant heating rate of 5 °C per minute. Data acquisition and analysis were performed using Calisto 2.0 software. For each composition, the experiment was repeated three times to ensure accuracy and reproducibility.

#### 3.5.4. Combustion Characteristics of Polyurethane Foams

A cone calorimeter test was conducted to evaluate the combustion properties of diarylheptanoid-based PUR foam compositions compared to those of the reference PUR foam composition. The reaction to fire performance was assessed using an FTT Dual Cone Calorimeter in accordance with ISO 5660-1:2015. The PUR foam specimens with dimensions of 100 × 100 × 24 mm were exposed to a constant heat flux of 35 kW/m^2^ to achieve ignition. The test duration was 300 s, and each composition was tested in triplicate to ensure reproducibility.

### 3.6. Thermal Oxidative Aging of Polyurethane Foams

The effect of oregonin-enriched extracts as an antioxidative on the color change in the PUR foams according to the results of accelerated thermal oxidative aging was studied. Composition for testing of Elastopir rigid polyurethanes (PUR) foam systems based on polyisocyanurate developed by BASF (Table 7) was kindly supplied by Latvian Ltd. TENAX PANEL (Dobele, Latvia).

The antioxidants, including oregonin-enriched extractives and Irganox 1010, in amounts of 1% and 2% of the total weight of the compositions were introduced into the polyol component, followed by premixing for 10 s. Subsequently, other components were added, and foaming was carried out as described in Section 3.4. After 24 h of post-curing the PUR foam at room temperature, cubic samples measuring 30 mm were cut and used for testing. The initial, extractive-free and Irganox-free PUR foam was used as the reference composition (Table 6).

Accelerated aging was carried out in oven at 150 °C during 24 and 48 h with constant air [41]. The PUR foams, both before and after aging, were analyzed using solid-phase UV-visible spectroscopy in the wavelength range of 190–800 nm employing a PerkinElmer UV-Vis spectrometer Lambda 650 with a 150 mm integrating sphere. In all tests, a white surface with 100% reflectance was used as a blank.

To quantify the extent of PUR foam discoloring, the increment of area under the UV-visible curve for the aged samples is expressed in percent relative to that of the sample before aging (2).

(2) $$∆S=\frac{S_{T}-S_{0}}{S_{0}}\times 100$$

The notations are listed as follows:

ΔS—the increment of area (%);
S_T_—the area under the UV-visible spectra curve of PUR foam after aging at 150 °C for 24 or 48 h, dimensionless (AU);
S_0_—the area under the UV-visible spectra curve of PUR foam before aging, dimensionless (AU).

## 4. Conclusions

This study underscores the potential of a black alder bark as a renewable and sustainable source of diarylheptanoids, aromatic secondary metabolites that serve as promising multifunctional components for polyurethane (PU) compositions.

The predominant black alder bark diarylheptanoid glucosides featuring catechol moieties connected by a seven-carbon chain, incorporating sugar units, and exhibiting high hydroxyl group functionality have been shown to effectively crosslink PU networks. Moreover, when introduced into a polyurethane matrix, the heptane chain of oregonin can act as a flexible segment, allowing for balance between rigidity and flexibility in the material, thereby improving its performance properties. These unique structural characteristics make diarylheptanoid glucosides advantageous building blocks for producing rigid polyurethane foams. In contrast, the widely studied diarylheptanoid curcumin, with its two aromatic ortho-methoxyphenol moieties and an α,β-unsaturated β-diketone group, offers comparatively limited functionality in PU systems.

The PUR foams incorporating black alder bark diarylheptanoid derivatives, as complete replacements for commercial polyols, exhibited significantly enhanced compression characteristics compared to those of the foams derived solely from fossil-based building blocks. Additionally, these biomass-based polyol-derived-PUR foams demonstrated superior thermal stability, as reflected by reduced weight loss during combustion and greater char retention at elevated temperatures. They also exhibited lower heat release rates, reduced smoke emissions, and extended flame-burning times, making them safer alternatives for applications with stringent fire safety requirements. To prevent the potential negative effects of excessively high functionality in oregonin-based polyols on certain PUR foam properties, particularly increased friability, their functionality can be carefully adjusted during the liquefaction process.

Diarylheptanoid glucosides, particularly those in oregonin-enriched black alder bark extracts, also demonstrated significant potential as natural phenolic antioxidants in PUR foams. During thermo-oxidative aging, their antioxidative effects, evidenced by reduced material darkening, surpassed those of the commercial antioxidant Irganox. This can be explained by the lower-level reactivity of the phenolic groups with isocyanate compared to that of the aliphatic hydroxyl groups, resulting in the partial retention of free phenolic groups in oregonin within the cured polyurethane matrix.

Furthermore, black alder bark diarylheptanoid glucosides, when used as building blocks in PU films, were found to be biodegradable by soil microorganisms. This bio-degradability is attributed to the presence of catechol moieties and the hydrolysis of glucosidic bonds in their structure, which facilitate degradation. In contrast, the aromatic moieties introduced by curcumin into the PU matrix are not biodegradable, highlighting an advantage of alder bark-derived compounds.

To summarize, alder bark represents a versatile, sustainable, and renewable source of diarylheptanoids with broad applicability in polyurethane materials. These compounds enhance mechanical strength, thermal stability, and fire resistance, while offering additional environmental benefits through biodegradability and antioxidative properties. Future research should focus on optimizing diarylheptanoid extraction processes and scaling up their industrial integration, further advancing their potential in eco-friendly polyurethane applications.

## Figures and Tables

**Table 1:** Characterization of hydrolyzed alder bark (*Alnus glutinosa*) extracts.

Extract	Yield (%)		Content in Initial Extract/Sugar-Free Hydrolysate (%)		
	On Extract	On Sugar-Free Hydrolysate	Oregonin	Hirsutenone	Xylose-Free Oregonin Analogue ^1^
Initial	-	76.6	71.4	-	-
Hydrolyzed_0h	92.9	70.8	24.1	28.6	1.5
Hydrolyzed_1h	96.2	83.3	8.8	34.4	22.9
Hydrolyzed_3h	98.7	71.9	0.3	25	16.3
Hydrolyzed_6h	98	67.7	0.2	21.2	11.6

^1^ Quantified with hirsutenone calibration curve.

**Table 2:** Tensile characteristics of model PU films and their Tg depending on polyol used.

Polyol	OH Functionality	E ^1^ (MPa)	σ_max_^2^ (MPa)	σ_b_^3^ (MPa)	ε_b_^4^ (%)	Τg ^5^ (°C)
Reference-PEG400	2	3.1 ± 0.3	12.9 ± 1.9	12.8 ± 1.9	335.2 ± 29	7.6
Oregonin-based polyol ^6^	7	2124 ± 186	49.1 ± 5.2	47.1 ± 5.2	3.6 ± 1.5	too high
Hirsutenone-based polyol ^7^	4	1046.1 ± 19.6	4.1 ± 1.0	4.1 ± 1.0	0.35 ± 0.07	82.7 ± 1.5
Curcumin-based polyol ^8^	3	426.4 ± 199.6	35.4 ± 0.33	27.8 ± 1.4	21.5 ± 3.3	38.0

^1^ E—Young’s modulus; ^2^ σ_max_—maximal stress; ^3^ σ_b_—stress at break; ^4^ ε_b_—elongation at break; ^5^ Tg—glass transition temperature. Extent of substitution of PEG 400 with biomass-based polyol, 81% ^6^, 64% ^7^; and 52% ^8^, amounting to 30% of biomass in PU.

**Table 3:** Relative content (%) ^1^ of compounds in pyrolysis products of PU films depending on polyol used before and after 60 days of biodegradation.

Groups of Compounds	Polyol Used			
	PEG400	Oregonin-Based Polyol ^2^	Hirsutenone-Based Polyol ^3^	Curcumin-Based Polyol ^4^
Acids, esters	3.4/2.9	0.3/0.3	0.5/0.1	2.6/2.7
Aldehydes, ketones	2.4/2.3	0/0	0/0	2.2/2.3
Aliphatic alcohols	47.9/49.6	2.9/2.8	4.3/0.9	22.1/16.9
Ethers	1.7/1.6	0/0	0/0	0.3/0.5
Furan derivatives	0/0	1.1/1.0	1.0/1.1	3.0/3.9
Cyclopentane derivates	0/0	0.2/0.2	0/0	0.3/0.1
Aromatic compounds	0.4/0.5	6.4/5.7	8.8/8.6	18.9/23.0
N-containing compounds	4.3/9.3	58.7/61.0	44.6/45.9	3.0/2.0

^1^—from sample chromatogram peak area before/after biodegradation. Extent of substitution of PEG 400 with biomass-based polyol, 81% ^2^, 64% ^3^; and 52% ^4^, amounting to 30% of biomass in PU.

**Table 4:** Key characteristics of biomass-based polyols used for polyurethane foam development.

Biomass-Based Polyol	OHV ^1^, mgKOH·g^−1^	Acid Value, mgKOH·g^−1^		MMD			Viscosity at 25 °C (mPa·s)
			H_2_O by K.F., % ^2^	Mn (Da)	Mw (Da)	D ^3^	
Liquefied oregonin-based polyol	539	0.35	0.25	n.d. ^4^	n.d. ^4^	n.d. ^4^	7350
Oxypropylated oregonin-based polyol	464	<LOD ^5^	0.09	2141	2414	1.13	1050

^1^—hydroxyl value; ^2^—Karl Fischer (K.F.) titration; ^3^—dispersity; ^4^—not determined; ^5^—lower than limit of detection (LOD) of titration method used.

**Table 5:** Mechanical performance of PUR foams depending on polyol used.

Polyol	Apparent Density (kg·m^3^)	Normalized Compression Strength * (MPa)	Normalized Compressive Young’s Modulus * (MPa)
Reference-Lupranol 3300	49 ± 1	0.18 ± 0.01	4.9 ± 0.4
Oxypropylated oregonin-based polyol	40.7 ± 0.6	0.31 ± 0.03	8.6 ± 0.7
Liquified oregonin-based polyol	60 ± 2	0.23 ± 0.02	6.7 ± 0.8

* Normalized to apparent density of 40 kg∙m^−3^ [32].

**Table 6:** Results of cone calorimetric combustion tests on PUR foams.

Parameters	Abbreviation	Dimension	Value	
			Reference PUR Foam	Biomass-Based Polyol-Derived PUR Foam
Time to ignition	TTI	s	6.7 ± 1.5	4.7 ± 1.1
Time to flameout	TTF	s	128.0 ± 14.9	172.7 ± 14.4
Mass losses	Δm	%	77.2 ± 2.7	79.6 ± 1.3
Specific mass loss rate 1 ^1^	Av MLR (*m_A_*_,0–50_)	g∙(s∙m^2^)^−1^	14.9 ± 0.2	10.7 ± 0.5
Specific mass loss rate 2 ^2^	Av MLR (*m_A_*_,10–75)_	g∙(s∙m^2^)^−1^	13.4 ± 0.5	9.0 ± 0.4
Total heat release	THR	MJ∙m^−2^	17.30 ± 0.81	19.70 ± 0.7
Average effective rate of combustion	Av-EHC	MJ∙kg^−1^	15.32 ± 0.39	17.16 ± 0.19
Average heat release rate	Av-HRR	kW∙m^−2^	59.2 ± 2.9	66.9 ± 2.0
Peak heat release rate	PHRR	kW∙m^−2^	276.3 ± 10.3	226.9 ± 7.6
Total smoke release	TSR	m^2^∙m^−2^	794.0 ± 20.5	644.5 ± 19.2
Maximum average rate of heat emission	MARHE	kW∙m^−2^	191.1 ± 1.7	142.0 ± 4.4
Average carbon dioxide yield	Av-CO_2_Y	kg∙kg^−1^	1.60 ± 0.03	1.75 ± 0.10
Average carbon monoxide yield	Av-COY	kg∙kg^−1^	0.087 ± 0.003	0.102 ± 0.002

^1^ Average specimen loss rate per unit area of sample between times of ignition and 50% of mass loss. ^2^ Average specimen loss rate per unit area of sample between 10% and 75% of mass loss.

**Table 7:** Composition of Elastopir 1132/509/0.

Component	Quantity	Unit
Polyol	100	p.b.w
Catalyst KX136	2.60	p.b.w
Catalyst KX 340/1	1.700	p.b.w
Blowing agent 96200	11.00	p.b.w
Isocyanate component	230	p.b.w

## Data Availability

The original contributions presented in this study are included in the article. Further inquiries can be directed to the corresponding author.

## Abbreviations

The following abbreviations are used in this manuscript:

PU	Polyurethane
PUR foam	Rigid Polyurethane Foam
FTIR	Fourier-Transform Infrared Spectroscopy
Py-GC-MS/FID	Pyrolysis-Gas Chromatography-Mass Spectrometry/Flame Ionization Detection
THF	Tetrahydrofuran
UPLC-ToF-MS	Ultra-Performance Liquid Chromatography–Time-of-Flight Mass Spectrometry.
UPLC-UV	Ultra-Performance Liquid Chromatography–Ultraviolet Detection
DABCO	1,4-diazabicyclo[2.2.2]octane
PEG400	Polyethylene glycol with the average molecular weight of 400 g/mol
UHLPC-ELSD	Ultra-High-Performance Liquid Chromatography with Evaporative Light Scattering Detection
TTI	Time of ignition
TTF	Time of flameout
THR	Total heat release
Av-HRR	Average heat release rate
PHRR	Peak heat release rate
Av-HER	Average effective rate of combustion
TSR	Total smoke release
MARHE	Maximum average rate of heat emission
UV-VIS	Ultraviolet-Visible Spectroscopy
OHV	Hydroxyl Value
PMDI	Polymeric diphenylmethane diisocyanate
PC	Propylene carbonate
